# Bibliometric insights into the most influential papers on antibiotic adjuvants: a comprehensive analysis

**DOI:** 10.3389/fphar.2023.1276018

**Published:** 2023-11-13

**Authors:** Ke Sun, Ping Xu, Yu Zhang, Pingjing Yu, Yuan Ju

**Affiliations:** ^1^ State Key Laboratory of Biotherapy and Cancer Center, Med-X Center for Manufacturing, Department of Laboratory Medicine, West China Hospital, Sichuan University, Chengdu, China; ^2^ Sichuan University Library, Sichuan University, Chengdu, China

**Keywords:** antibiotic adjuvants, bibliometric analysis, top-cited, citation analysis, VOSviewer

## Abstract

**Background:** The utilization of antibiotic adjuvants presents a promising strategy for addressing bacterial resistance. Recently, the development of antibiotic adjuvants has attracted considerable attention from researchers in academia and industry. This study aimed to identify the most influential publications on antibiotic adjuvants and elucidate the hotspots and research trends in this field.

**Method:** Original articles and reviews related to antibiotic adjuvants were retrieved from the Web of Science Core Collection database. The top 100 highly cited publications were selected and the visual analyses of publication outputs, countries, institutions, authors, journals, and keywords were conducted using Excel, VOSviewer, or CtieSpace software tools.

**Results:** The top 100 cited publications concerning antibiotic adjuvants spanned the years 1977–2020, with citation counts ranging from 174 to 2,735. These publications encompassed 49 original articles and 51 reviews. The journal “*Antimicrobial Agents and Chemotherapy*” accounted for the highest number of publications (12%). The top 100 cited publications emanated from 39 countries, with the United States leading in production. Institutions in Canada and the United States exhibited the most substantial contributions to these highly cited publications. A total of 526 authors participated in these studies, with Robert E.W. Hancock, Laura J. V. Piddock, Xian-Zhi Li, Hiroshi Nikaido, and Olga Lomovskaya emerging as the most frequently nominated authors. The most common keywords included “*E. coli*”, “*P. aeruginosa*”, “*S. aureus*”, “*in-vitro* activity”, “antimicrobial peptide”, “efflux pump inhibitor” “efflux pump”, “MexAB-OprM” and “mechanism”. These keywords underscored the hotspots of bacterial resistance mechanisms and the development of novel antibiotic adjuvants.

**Conclusion:** Through the bibliometric analysis, this study identified the top 100 highly cited publications on antibiotic adjuvants. Moreover, the findings offered a comprehensive understanding of the characteristics and frontiers in this field.

## 1 Introduction

Since Alexander Fleming discovered penicillin in 1928, the advent of antibiotics marked a “golden age” from the 1940s to the 1960s (Hutchings et al., 2019). Antibiotics have saved countless lives, and they remain indispensable tools in preventing infections during surgical procedures, organ transplants, cancer chemotherapy, etc. However, antibiotic resistance is on the rise due to their overuse and misuse in healthcare and agriculture. Antibiotic resistance has become a formidable challenge to public health in the 21st century (Byrne et al., 2019). Notably, ESKAPE pathogens (*Enterococcus faecium*, *Staphylococcus aureus* (*S. aureus*), *Klebsiella pneumoniae*, *Acinetobacter baumannii*, *Pseudomonas aeruginosa* (*P. aeruginosa*), and *Enterobacter species*) pose substantial threats due to their extensive resistance to the first line and last-resort antibiotics (Boucher et al., 2009). More urgently, there have been ever fewer new antibiotics available for clinical use. Pharmaceutical companies are reluctant to invest in the development of antibiotics because of the low financial returns (Plackett, 2020). Overcoming antibiotic resistance presents a formidable task. There is an urgent need to develop alternative strategies that can curtail the emergence of antibiotic resistance or extend the effectiveness of existing (Tyers and Wright, 2019). Combining antibiotic adjuvants with an antibiotic offers one such approach to combat antibiotic-resistant bacteria.

Antibiotic combinations (antibiotic A + antibiotic B) sometimes manifest synergistic actions by acting on a variety of mechanisms, including targeting different steps in a common or related biochemical pathway (Worthington and Melander, 2013). This generally makes antibiotics more effective than the sum of individual agents. As a result, it leads to faster kill times or enhanced killing, thereby limiting the opportunity for resistant organisms to emerge. Distinctively, antibiotic adjuvants are often delivered together with a known antibiotic, but they have no or little antibacterial activity alone (Worthington and Melander, 2013; Wright, 2016). These adjuvants act to suppress active or passive resistance elements in bacteria or enhance host response. Thereby, they offer the potential to reinvigorate the efficacy of antibiotics against resistant bacteria. Furthermore, antibiotic adjuvants can also expand the antibiotic spectrum and reduce the required dose of antibiotics (Wright, 2016). Antibiotic adjuvants can also be referred to as “chemosensitizers” or “antibiotic potentiators” (Yates and Stephenson, 1960; Gallo, 2003). Currently, β-lactamase inhibitors, a type of antibiotic adjuvant, have been clinically approved for combined use with β-lactam antibiotics. Meanwhile, several other antibiotic-adjuvant combinations are undergoing clinical trials (Dhanda et al., 2023). The success of these approaches has inspired researchers to explore other types of antibiotic adjuvants and catalyzed plenty of attention to this field in recent years.

Bibliometric analysis stands as a popular and powerful tool for both basic science researchers and clinical workers to obtain comprehensive overviews of certain research fields (García-Fernndez et al., 2020). Citation analysis is a method of bibliometric analysis that purposed to identify studies frequently cited by authors and their peers (Aslam-Pervez and Lubek, 2018; Arshad et al., 2020). In particular, reviewing the “highly cited” or “top-cited” publications facilitates the identification of scientific excellence, outstanding authors or institution impacts, and discerning research hotspots (Jenkins et al., 2022; Li et al., 2022). Bibliometric analysis of the top 100 most-cited publications has offered insights into research related to antibacterial compounds, including dalbavancin (Monteagudo-Martínez et al., 2022), bacteriocins (Jamali et al., 2019), and antibiotics (Arshad et al., 2020). Although a bibliometric analysis has analyzed clavulanic acid-related studies (Ramirez-Malule, 2018), there is no citation analysis of the top-cited publications on antibiotic adjuvants. This study was proposed to identify and discuss the top 100 cited publications concerning antibiotic adjuvants. The results will provide basic science researchers and clinical workers with insights into pivotal research strides and noteworthy advancements of this field.

## 2 Materials and methods

### 2.1 Data sources

The publications for this study were sourced from the Web of Science Core Collection (WosCC) database from its inception up to 1 March 2023 (updated until 3 April 2023). The advanced search strategy was employed as follows: TS=(((antibiotic* or antimicrobial or antibacterial or Gram) NEAR/6 (adjuvant$ or potentiator$ or synerg*)) or chemosensitizer$ or membrane permeabilizer$ or (efflux Pump$ NEAR/5 inhibit*) or (quorum sensing NEAR/5 inhibit$) or (bate-Lactamase NEAR/5 inhibit*) or clavulanic acid or clavulanate or sulbactam or tazobactam or ((immun* NEAR/4 (stimulant$ or augment$ or activat$ or potentiat$ or modulat$)) NEAR/5 (bacter* or microbi* or antimicrobe* or antibacter*))). The asterisk (*) represents any group of characters, including no character. The dollar sign ($) represents zero or one character. The NEAR/5 or NEAR/4 finds records where the terms joined by the operator are within 5 or 4 words of each other, and the number 5 or 4 can specify the maximum number of 5 or 4 words that separate the terms. In total, 30,557 publications were retrieved, and subsequent refinement was performed to include only original articles or reviews. Additionally, the publications were ranked in descending order according to the citation counts. To ensure precision, two authors (Yu Zhang and Pingjing Yu) conducted a manual assessment. They reviewed the abstracts or full texts to exclude publications that were not relevant to antibiotic adjuvants. This process ultimately yielded the definitive selection of the top 100 highly cited publications and Supplementary Table S1 listed these influential publications.

### 2.2 Data extraction

Full records of the top 100 highly cited publications were downloaded from the WoSCC database, including authors, title, journal, publication year, citation counts in all databases, addresses, affiliations, document type, keywords, and WoS Categories. The impact factors (IFs) and quartiles in category of journals were ascertained from the Journal Citation Reports (JCR, https://jcr.clarivate.com/) for the year 2022. Some regions of certain countries are presented separately in the publication, while they are generally regarded as one country internationally. Therefore, we performed a data refinement process to combine Scotland and England into the United Kingdom and combine Taiwan into China. In addition, merging keywords with similar meanings could help normalize different forms of text words, and then correctly calculate the occurrence frequency of keywords and generate the co-occurrence network of keywords. In addition, authors with the same name were authenticated through cross-referencing authors’ affiliations and collaborators.

### 2.3 Data analysis

Data were downloaded as Excel file or text file. The text file was imported into VOSviewer and Citespace, which are commonly used and popular software tools for bibliometric network creation and visualization (Chen, 2006; van Eck and Waltman, 2010). Microsoft Excel 2010 was used for statistical analysis of publication year distribution, publication type, citation count ranking of author/country/institution/journal, and the journal’s IF. The correlation coefficient, including the Spearman rank test and Kruskal-Wallis test, was conducted using IBM SPSS Statistics 26. Subsequently, VOSviewer 1.6.17, Citespace 6.2. R4, and Scimago Graphica were employed to obtain visualization and bibliometric maps. We conducted analyses of citation and co-citation. The co-occurrence networks of keywords, authors, and countries were visualized via VOSviewer 1.6.17. The counting method used in the VOSviewer analysis was full counting. The Co-cited references map was generated using Citespace 6.1. R6 (Basic). The CiteSpace parameters used for analysis were as follows: Link retaining factor of 3.0; Time span (1977–2020) with one slice per year; Node types (Reference); Links (strength: cosine, scope: within slices); Selection criteria (g-index scale factor of 10).

## 3 Results

### 3.1 Analysis of publication years and types

**FIGURE 1:**
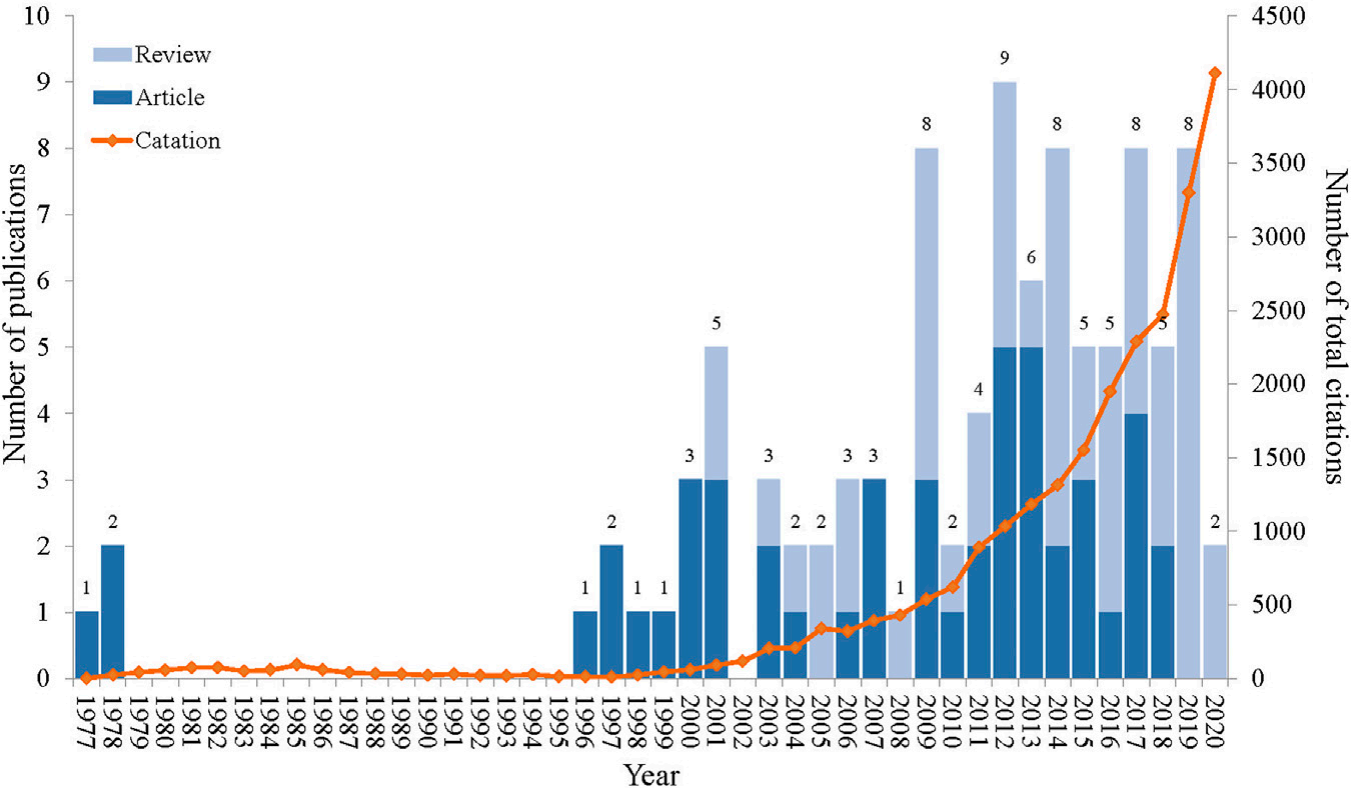
Annual trends in the number of publications and citation counts related to the top 100 cited publications. The numbers at the top of the column indicated the annual number of publications; the light blue column represented the proportion of reviews; the dark blue column represented the proportion of articles; and the orange line represented per year total citation counts.

The top 100 cited papers were published from 1977 to 2020, with an absence of highly cited articles between 1979 and 1995 (Figure 1). Among the publication years, 2012 stood out with the highest number of publications (9), followed by 8 papers each in 2009, 2014, 2017, and 2019. The citation counts of these publications ranged from 174 to 2,735 times, and 4 publications accumulated more than 1,000 times. Notably, the citation counts exhibited a progressive upward trajectory over the years (Figure 1). The most cited paper was published by Michael R. Yeaman and Nannette Y. Yount in 2003, involving the potential therapeutic applications of antimicrobial peptides (AMPs) to enhance the antibacterial effect of traditional antibiotics (Yeaman and Yount, 2003). Moreover, the most recent paper was published on 16 March 2020, with citation counts of 353 times. This publication reviewed antibiotic adjuvants as novel treatments against resistant Gram-negative bacteria (Breijyeh et al., 2020). The Kruskal-Wallis tests were performed to find the variation in citation counts across publication years, but no statistically significant difference (test statistic (*X* ^2^) = 43.00, *p* = 0.462) was observed.

The list of the top 100 cited publications encompassed 49 original articles and 51 reviews. In the earlier stage (1977–2000), there were all original articles (Figure 1). These articles revolved around clavulanic acid, including the kinetics of inhibiting β-lactamase by clavulanate acid and the antibacterial effect of amoxicillin-clavulanate (Reading and Cole, 1977; Fisher et al., 1978; Andes and Craig, 1998). Novel peptides, membrane permeabilizers, efflux pump inhibitors (EPIs), and plant compounds were also explored to enhance the activity of antibiotics (Vaara and Porro, 1996; Helander et al., 1997; Renau et al., 1999; Stermitz et al., 2000). Although this initial phase only featured 12 publications in the top 100 cited list, these works laid the theoretical basis for subsequent explorations in the field of antibiotic adjuvants. Notably, the highly cited publications in 2019 and 2020 were exclusively constituted by reviews. This was possibly due to the inherent tendency of reviews to accumulate more citation counts compared to original articles (Patsopoulos, 2005; Ioannidis et al., 2016).

### 3.2 Journals

The top 100 cited publications were from 64 journals and the 2022 IFs ranged from 2.2 (*American Journal of Translational Research*) to 120.1 (*Nature Reviews Drug Discovery*). Notably, 62 papers were from journals with 2022 IFs surpassing 5. Among them, 36 papers were from journals with 2022 IFs exceeding 10. It was noteworthy that *Nanoscale Research Letters* and *Chemistry* & *Biology* were not included in the 2022 edition of JCR. Dominantly, the top 100 cited publications were from journals encompassing domains of microbiology, pharmacology, and chemistry. The Spearman rank test indicated that the number of publications (r = 0.267, *p* = 0.036) and the total citation counts of journals (r = 0.391, *p* = 0.002) had positive correlations to the 2022 IFs of journals.

Table 1 listed the journals that published more than 1 highly cited publication, and these journals have been stratified into Q1 or Q2 categories. The *Antimicrobial Agents and Chemotherapy* (2022 IF = 4.9) emerged as a pivotal player with the largest number of publications (12, 12%). This journal mainly publishes original articles from areas of antibacterial, antiviral, antifungal, and antiparasitic agents. Earlier research on clavulanic acid was published in the *Antimicrobial Agents and Chemotherapy*, and this journal is also an important platform for antimicrobial research (Reading and Cole, 1977; Neu and Fu, 1978). The *Clinical Microbiology Reviews* (2022 IF = 36.8) and *Drugs* (2022 IF = 11.5) ranked second with 4 publications each, and the *Clinical Microbiology Reviews* outperformed in total citation counts (3,194). This might be because the reviews in the *Clinical Microbiology Reviews* covered a wide range of topics, including the novel strategy of phage-antibiotic combinations, the development of β-lactamase inhibitors, and the progress of EPIs (Drawz and Bonomo, 2010; Li et al., 2015; Gordillo Altamirano and Barr, 2019).

**TABLE 1:** Journals contributing to the top 100 cited publications on antibiotic adjuvants (number of articles ≥2).

Journal	Number of publication	Total citation counts	2022 IF	Quartile in category
Antimicrobial Agents and Chemotherapy	12	4,619	4.9	Microbiology (Q1); Pharmacology & Pharmacy (Q2)
Clinical Microbiology Reviews	4	3,194	36.8	Microbiology (Q1)
Drugs	4	1,678	11.5	Pharmacology & Pharmacy (Q1); Toxicology (Q1)
Proceedings of the National Academy of Sciences of the United States of America	3	1,357	11.1	Multidisciplinary Sciences (Q1)
Nature Reviews Microbiology	3	946	88.1	Microbiology (Q1)
Natural Product Reports	3	754	11.9	Biochemistry & Molecular Biology (Q1); Chemistry, Medicinal (Q1); Chemistry, Organic (Q1)
Frontiers in Microbiology	2	958	5.2	Microbiology (Q2)
Phytomedicine	3	966	7.9	Chemistry, Medicinal (Q1); Integrative & Complementary Medicine (Q1); Pharmacology & Pharmacy (Q1); Plant Sciences (Q1)
Molecules	2	758	4.6	Biochemistry & Molecular Biology (Q2); Chemistry, Multidisciplinary (Q2)
Science Translational Medicine	2	697	17.1	Cell Biology (Q1); Medicine, Research & Experimental (Q1)
Journal of Antimicrobial Chemotherapy	2	579	5.2	Infectious Diseases (Q2); Microbiology (Q2); Pharmacology & Pharmacy (Q1)
Advanced Drug Delivery Reviews	2	550	16.1	Pharmacology & Pharmacy (Q1)
Critical Reviews in Microbiology	2	519	6.5	Microbiology (Q1)
Microbiological Research	2	487	6.7	Microbiology (Q1)
International Journal of Nanomedicine	2	407	8	Nanoscience & Nanotechnology (Q2); Pharmacology & Pharmacy (Q1)
Nature Microbiology	2	395	28.3	Microbiology (Q1)
Journal of Biological Chemistry	2	367	4.8	Biochemistry & Molecular Biology (Q2)
Plos One	2	365	3.7	Multidisciplinary Sciences (Q2)

### 3.3 Countries

**FIGURE 2:**
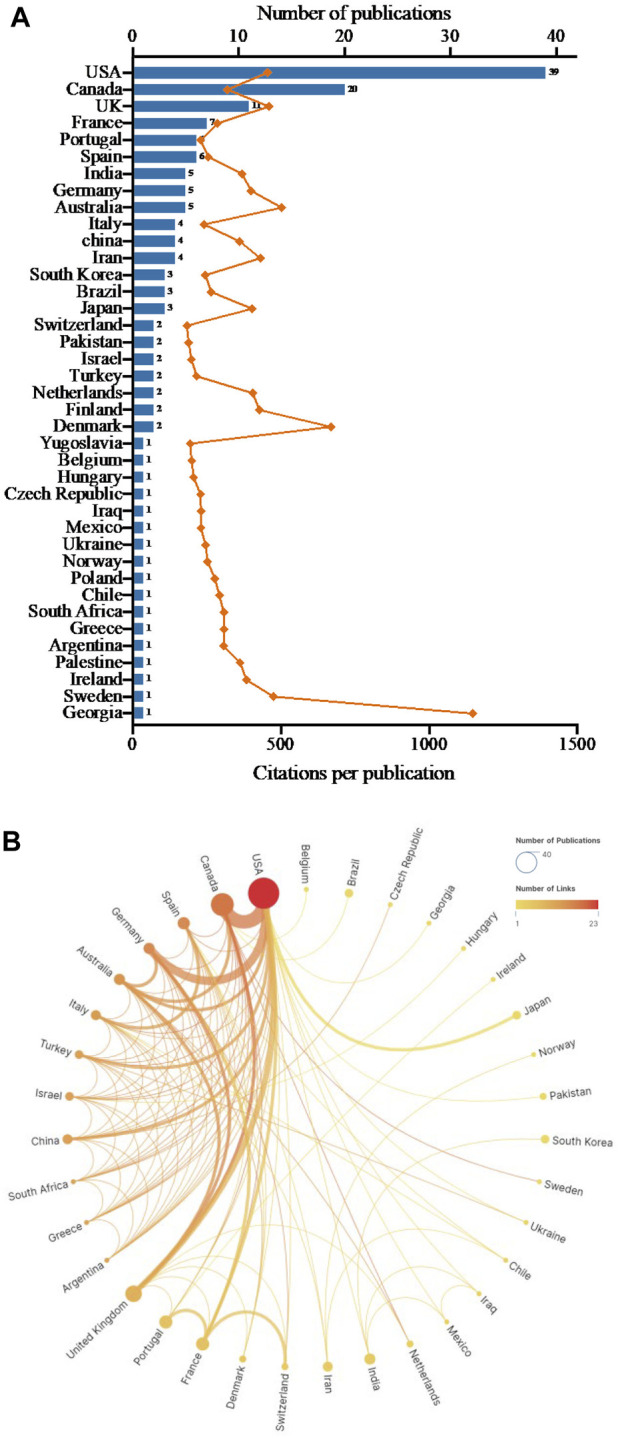
Countries contributed to the top 100 cited publications on antibiotic adjuvants. **(A)** The number of publications and citation counts from each of those countries. The column represented the number of publications per country, and the orange line represented the average number of citation counts per country. **(B)** International cooperation networks between countries. The size of the circles was proportional to each country’s publication count, the color of the circles depicted the extent of linkages, and the thickness of the connecting lines signaled collaboration frequency.

A total of 39 countries contributed to the top 100 highly cited publications in the field of antibiotic adjuvants (Figure 2A). Among them, the United States (n = 39), Canada (n = 20) and the United Kingdom (n = 11) emerged as the most prolific contributors with more than 10 papers each. Following closely were France (n = 7), Portugal (n = 6), and Spain (n = 6). Remarkably, publications from Georgia exhibited notable influence with an average citation count per publication of 1,147 times, followed by Denmark (669), Australia (502), Sweden (475), United Kingdom (460), and United States (455) (Figure 2A). Notably, initial papers from the earlier period (1977–1978) primarily originated from the United States and United Kingdom. This signified the pioneering roles of these countries in the field of antibiotic adjuvants. Between 1998 and 2019, the United States consistently generated highly cited publications. This indicated that the United States was ongoing concerned about the treatment of resistant bacteria and the development of antibiotic adjuvants.

The degree of collaboration (DC) between countries was 0.34. Among them, Finland, Ireland, Palestine, Yugoslavia, and Poland independently contributed 1 or 2 papers, and they did not participate in international collaborations. Figure 2B showed the international cooperation between countries. The results showed that the United States had the highest number of links with other countries and cooperated most frequently with Canada. Additionally, Spain, Germany, Australia, Italy, Israel, Greece, and United Kingdom in Europe manifested robust networks of cooperation among themselves.

### 3.4 Institutions

For the bibliometric analysis, a total of 177 institutions contributed to the top 100 cited publications on antibiotic adjuvants. Among these, 31 institutions yielded a minimum of 2 papers (Table 2). A significant portion of these research entities was affiliated with the United States (11), followed by Canada with 5 institutions, while United Kingdom, Portugal, and India each boasted 2 institutions. McMaster University in Canada was the most productive institution with 6 papers. In addition, the University of California, Los Angeles garnered the highest citation counts (2,845). It was worth to be noted that several pharmaceutical companies contributed to the development of novel antibiotic adjuvants. For example, the Microcide Pharmaceuticals Company and Merck & Company from the United States, as well as the Daiichi Sankyo Company Limited from Japan, each produced 2 publications. These companies collaborated with universities (like Merck & Company and Universidade Nova de Lisboa) to develop novel inhibitors as antibiotic adjuvants (Toney et al., 2001; Tan et al., 2012) or collaborated with one another (like Microcide Pharmaceuticals Company and Daiichi Sankyo Company Limited) to identify EPIs in *P*. *aeruginosa* (Renau et al., 1999; Lomovskaya et al., 2001). Beyond these, a range of other pharmaceutical companies also emerged as contributors to antibiotic adjuvant research, including Hunan Beautide Pharmaceut, AstraZeneca, Mpex Pharmaceuticals, and GlaxoSmithKline. Their publications delved into themes such as β-lactamase inhibitors, EPIs, or AMPs as adjuvants (Reading and Cole, 1977; Lomovskaya and Bostian, 2006; Ehmann et al., 2012; Lei et al., 2019).

**TABLE 2:** Institutions contributing to the top 100 cited publications on antibiotic adjuvants (number of articles ≥2).

Rank^a^	Institution	Number of publication	Total citation counts	Country
1	McMaster University	6	1,443	Canada
2	University of Birmingham	5	1769	England
3	University of British Columbia	5	1,694	Canada
4	University of Manitoba	4	1,173	Canada
5	Aix-Marseille Universite	4	784	France
6	Universidade Nova de Lisboa	4	384	Portugal
7	University of California, Los Angeles	3	2,845	United States
8	University of California, Berkeley	3	2,295	United States
9	Case Western Reserve University	3	1848	United States
10	Louis Stokes Cleveland Veterans Affairs Medical Center	3	1848	United States
11	Harvard University	3	1,094	United States
12	Health Canada	2	1,469	Canada
13	University System of Maryland	2	1,398	United States
14	University of Copenhagen	2	1,338	Denmark
15	Tabriz University of Medical Science	2	1,134	Iran
16	Colorado State University	2	921	United States
17	Microcide Pharmaceuticals Inc	2	908	United States
18	Daiichi Sankyo Company Limited	2	908	Japan
19	Boston University	2	889	United States
20	VTT Technical Research Center Finland	2	856	Finland
21	Utrecht University	2	810	Netherlands
22	Savitribai Phule Pune University	2	791	India
23	Indian Institute of Technology	2	744	India
24	University of London	2	654	England
25	Universidade do Porto	2	568	Portugal
26	Wayne State University	2	513	United States
27	Hospital Universitari Son Espases	2	507	Spain
28	Merck & Company	2	369	United States
29	University of Toronto	2	356	Canada
30	University of Queensland	2	249	Australia
31	University of Bern	2	192	Switzerland

^a^ The institutions were ranked by the number of publications.

### 3.5 Authors

There were 526 authors who contributed to the top 100 cited publications. Among them, 40 authors were featured in more than one paper. The average number of authors per publication stood at 5.77. These authors showed a high degree of collaboration, and only 4 publications showcased a single author. Furthermore, 23 publications featured two authors, while 21 publications enlisted three authors, and 52 papers embraced four or more authors. The apex of co-authors was marked by 2 papers featuring 38authors. These two articles involved the pioneering research to identify novel adjuvants, and the cohort study to investigate the effectiveness of β-lactam/β-lactamase inhibitor (Tan et al., 2012; Gutiérrez-Gutiérrez et al., 2016). Authors who contributed three or more papers were presented in Table 3. These productive authors predominantly occupied roles as first or corresponding authors. Robert E.W. Hancock, affiliated with the University of British Columbia, stood as the most prolific contributor with 5 papers. His research interests included the development of AMPs as antibiotic adjuvants against biofilms and infections (Yeung et al., 2011; Reffuveille et al., 2014; de la Fuente-Núñez et al., 2015). Laura J. V. Piddock from the University of Birmingham and Gerard D. Wright from McMaster University both ranked second with 4 papers. Laura J. V. Piddock focused on drug combinations (Ejim et al., 2011; Tyers and Wright, 2019). Gerard D. Wright was involved in efflux pumps (Piddock, 2006; Blair et al., 2014). Among the 7 productive authors with three or more publications, 3 authors were from the United States, 2 authors were from Canada1, and 1 author was each from United Kingdom and France. Michael R. Yeaman and Nannette Y. Yount from the University of California, Los Angeles were the most influential authors because they published the most cited paper with 2,253 citation counts (Yeaman and Yount, 2003).

**TABLE 3:** Authors contributing to the top 100 cited publications on antibiotic adjuvants (number of articles ≥3).

Author	Number of publication	First/Corre-sponding author	Total citation counts	Institution	Country
Robert E.W. Hancock	5	5	1,386	University of British Columbia	Canada
Laura J. V. Piddock	4	3	1,487	University of Birmingham	United Kingdom
Gerard D. Wright	4	4	1,052	McMaster University	Canada
Hiroshi Nikaido	3	3	1893	University of California, Berkeley	United States of America
Xian-Zhi Li	3	3	1893	Health Canada	Canada
Robert A. Bonomo	3	2	1758	Case Western Reserve University	United States
Olga Lomovskaya	3	2	1,145	Mpex Pharmaceut Inc	United States
Jean-Marie Pagès	3	2	581	Aix-Marseille Universite	France

**FIGURE 3:**
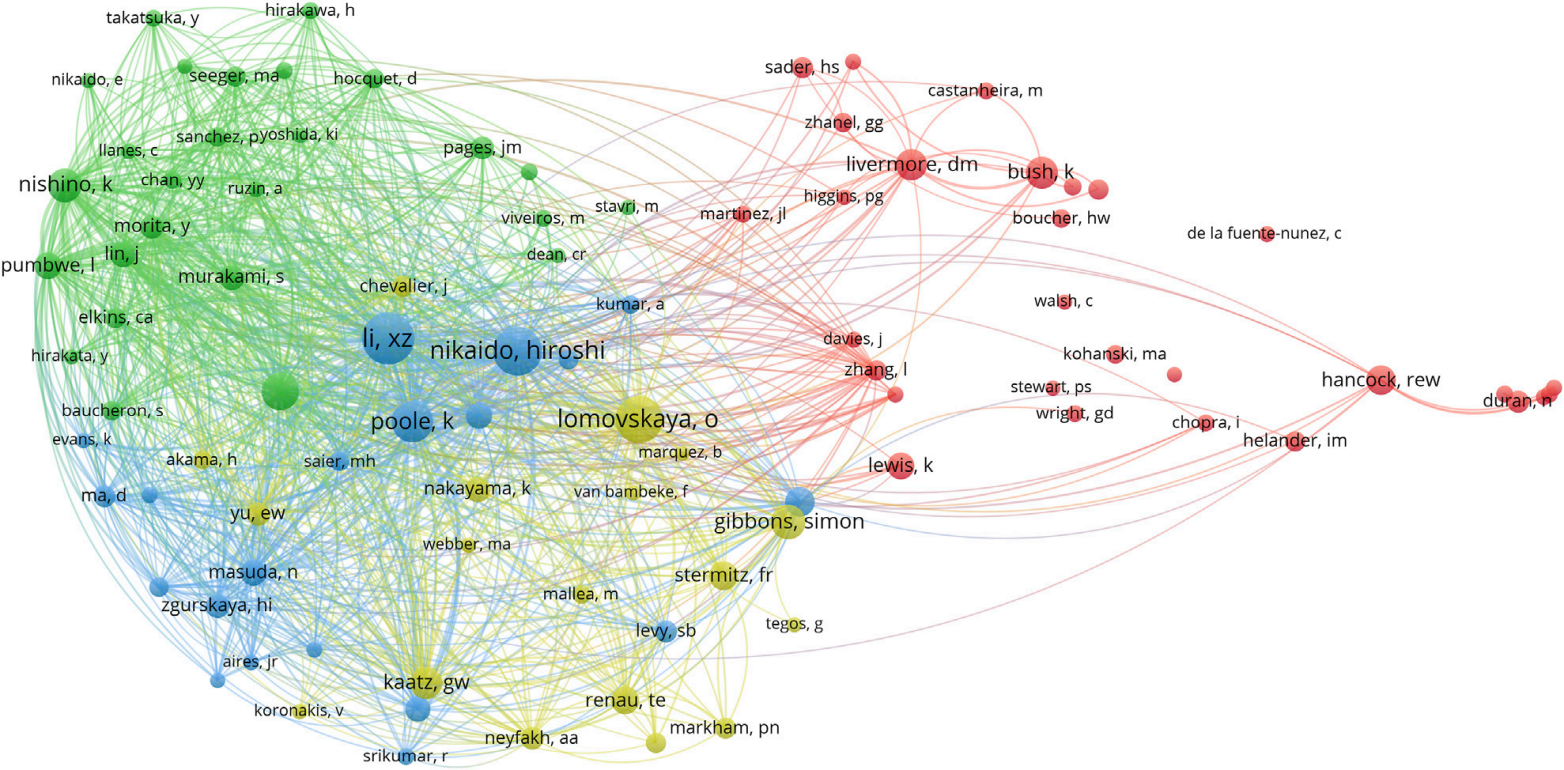
Co-cited authors of the top 100 cited publications on antibiotic adjuvants. Authors with citation counts at least 10 times were presented in the network. Each node represents an individual author and the size of the node was proportional to the citation count. The line between two nodes indicated these two authors were cited by one author and the line thickness represented the link strength between the authors (weighted by a quantitative evaluation indicator of TLS). Authors with close relationships were assigned to one cluster with the same color, and clusters of four colors (red, blue, green, yellow) represented four clusters.

To identify the research communities, the co-citation network of authors was constructed. This intricate network encompassed 6,752 co-cited authors, and 93 authors with the number of co-citations exceeding 10 were presented in Figure 3. The node sizes indicated the citation counts of authors, and these authors were stratified into four distinct clusters. Xian-Zhi Li from Health Canada (formerly the University of California, Berkeley) and Hiroshi Nikaido from the University of California, Berkeley were the most influential authors with citation counts of 80 times and 71 times, respectively. In addition, they had three co-published papers in the list of the top 100 cited publications. Moreover, Olga Lomovskaya (citation counts of 66 times) and Laura J. V. Piddock (citation counts of 44 times) also featured prominently within the category of prolific authors (Table 3). These authors also had the greatest total link strength (TLS). The top 6 authors with the greatest TLS were Xian-Zhi Li (TLS = 8,005), Hiroshi Nikaido (TLS = 5,841), Keith Poole (TLS = 4,899), Koki Nishino (TLS = 4,511), Laura J. V. Piddock (TLS = 4,179), and Olga Lomovskaya (TLS = 4,073). This indicated that Xian-Zhi Li (blue cluster), Hiroshi Nikaido (blue cluster), Laura J. V. Piddock (green cluster), and Olga Lomovskaya (yellow cluster) played pivotal roles in antibiotic adjuvants research. Moreover, Karen Bush from Indiana University (formerly Johnson & Johnson) claimed a paramount position within the red cluster with citation counts of 35 times and 834 TLS. She focused on the study of β-lactamase and novel antibacterial compounds (Baum et al., 2007; Queenan et al., 2007; Bush and Bradford, 2020).

### 3.6 Keywords

**FIGURE 4:**
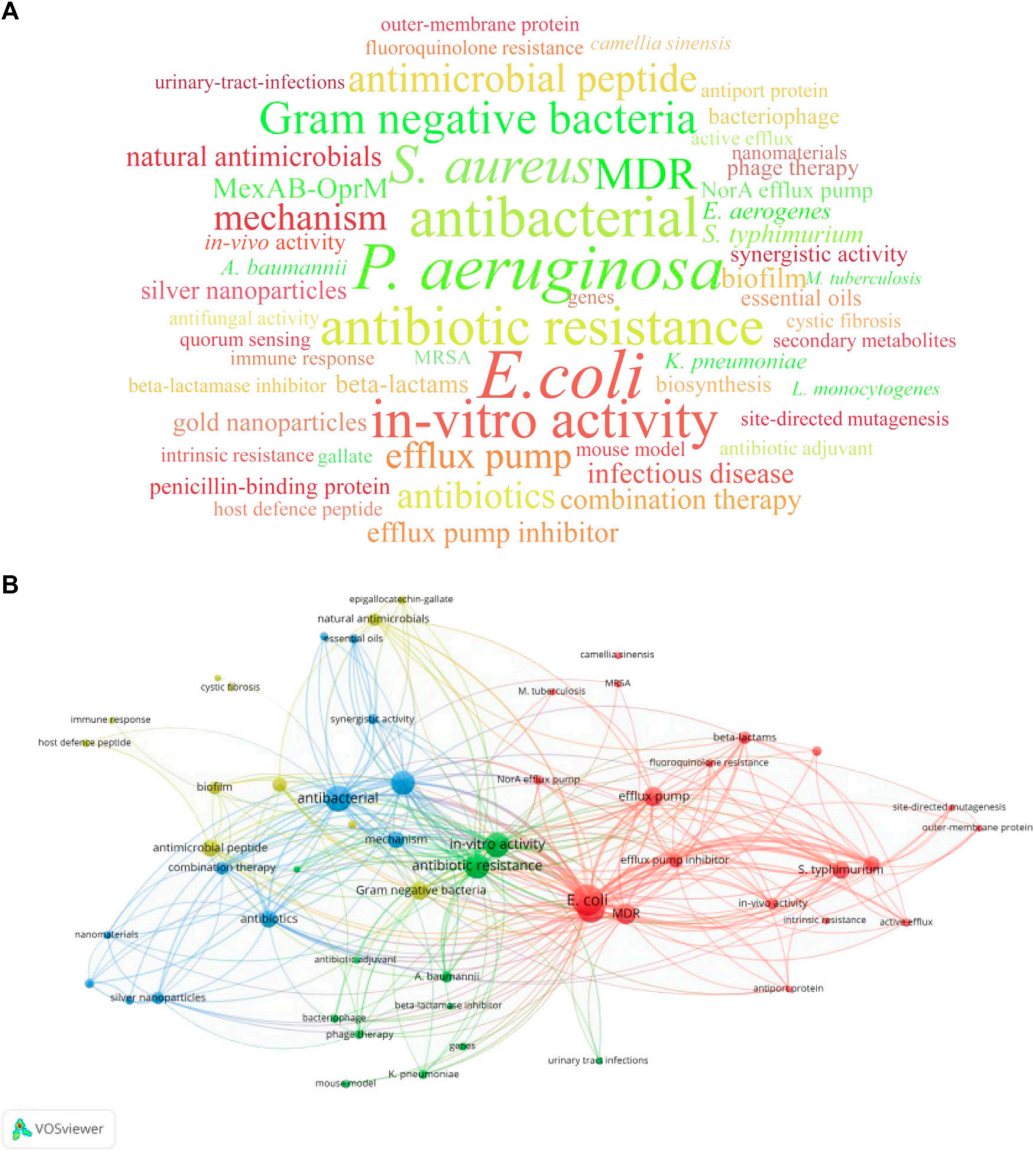
Keywords analysis of the top 100 cited publications related to antibiotic adjuvants. **(A)** Keywords cloud of keywords that occurred at least 3 times. The size of the word was proportional to the occurrence frequency. **(B)** Co-occurrence network of keywords with occurrence frequency more than 2 times. Each node represented a keyword and the size of the node was proportional to the occurrence frequency. The closely related keywords are clustered by color, and clusters of four colors (red, blue, green, yellow) represented four clusters.

It should be specified that 3 publications published before 1978 did not have author keywords and keywords plus, hence, they were excluded from the keyword analysis. A total of 332 keywords were extracted from the remaining 97 publications. Among them, 229 keywords appeared only once, accounting for a substantial 68.98% of the aggregate. Moreover, 89 keywords (26.80%) made appearances ranging between 2 and 10 times, while the remaining top 14 keywords (4.22%) featured over 10 times. The top 14 keywords included three bacterial strains: “*Escherichia coli* (*E. coli*)” (occurring 43 times), “*P. aeruginosa*” (occurring 35 times), and “*S. aureus*” (occurring 27 times). These strains have a high level of resistance to antibiotics and are classified as ESKAPE pathogens. Keywords related to anti-bacterial compounds appeared twice, including “antimicrobial peptide” (occurring 14 times) and “efflux pump inhibitor” (occurring 10 times). This revealed that these compounds were often used as antibiotic adjuvants against bacterial infections. Furthermore, subject “mechanism” occurred 14 times, and related targets “efflux pump” and “MexAB-OprM” also appeared over 10 times. Research methods, namely, “*in-vitro* activity” occurred 29 times. The co-occurrence network of keywords manifested for keywords appearing 3 or more times (Figure 4). Accordingly, these keywords were divided into four clusters: “efflux pump” (red), “nanomaterials” (blue), “beta-lactamase inhibitor” (green), and “antimicrobial peptide” (yellow).

### 3.7 Co-cited references

**FIGURE 5:**
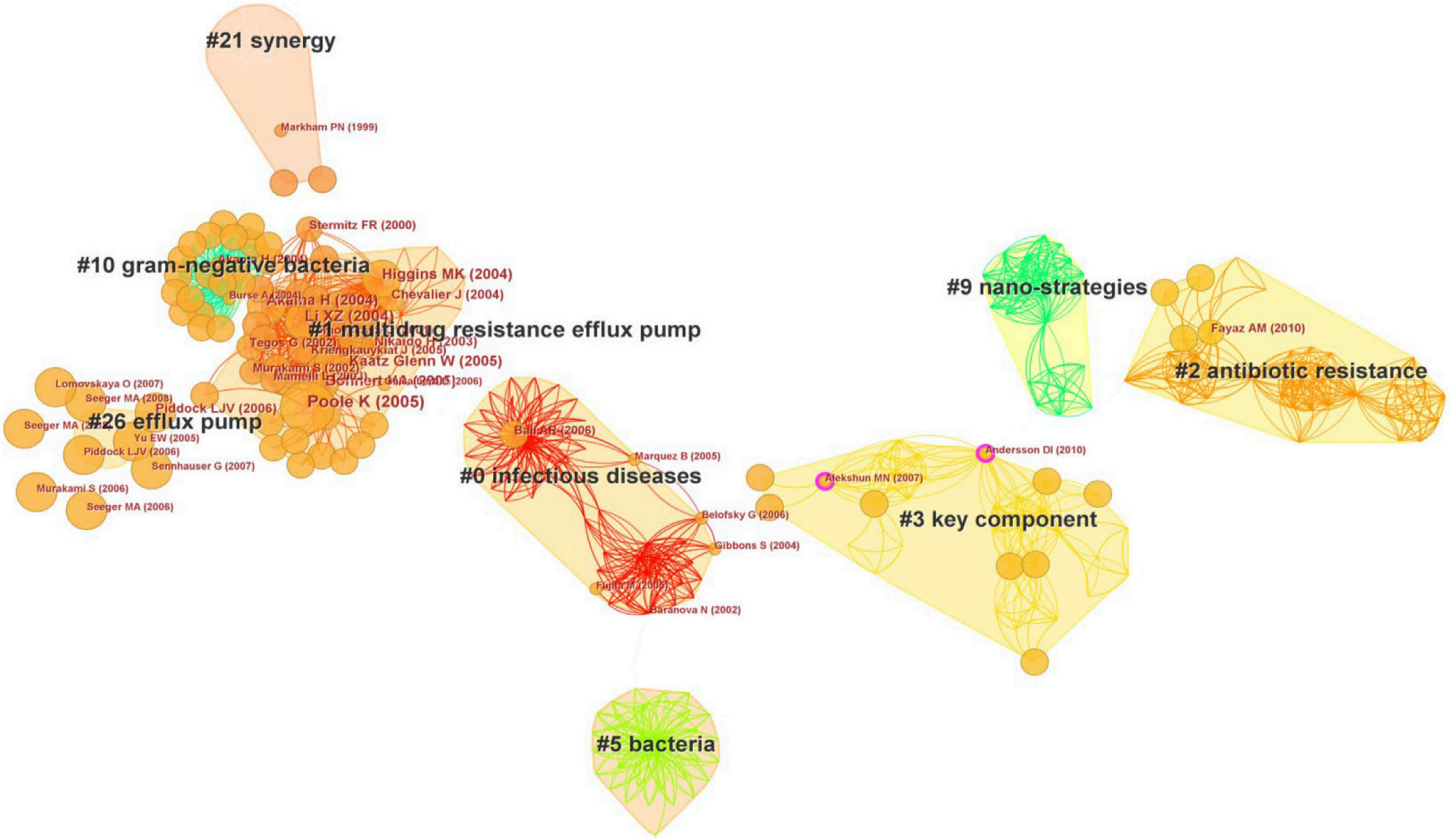
Co-cited references of the top 100 cited publications on antibiotic adjuvants. Each node represented a reference, and nodes in the same color block represented a cluster with the same topic. The smaller number of cluster indicated that the cluster contains more nodes. The name of the cluster was extracted from the titles of references in the cluster, and it represented the topics of the references. Cluster filters: show the largest connected component only; CiteSpace configuration: LRF = 3, LBY = 5, L/N = 5, e = 1.0, and g-index (k = 10). Network: 796 references and 5,182 co-citation links.

Co-citation is defined as the simultaneous citation of two documents in a single publication (Small, 1973). The map of co-cited references evaluates the scientific relevance of publications when they are frequently co-cited by other papers. Furthermore, co-citation clustering enables the delineation of the knowledge base and disciplinary structure of related research (Leung et al., 2017). The top 100 cited publications underwent a co-citation analysis facilitated by CiteSpace. The co-citation analysis yielded nine large clusters (Figure 5). These clusters were generated by the spectral clustering of Citespace software. A smaller number of cluster indicated that the cluster contains more nodes, where cluster #0 has the largest number of references. Since S < 0.5 represents incredible clustering, some numbers are missing as their S values are below 0.5. The modularity Q score was 0.933, and the Weighted Mean Silhouette S score amounted to 0.9685. These denoted a well-structured co-citation network with relatively minimal inter-cluster connections (Chen et al., 2010). Of these clusters, cluster #0 labeled as “infectious diseases” emerged as the largest cluster with 49 co-cited references. Notably, the most cited paper in cluster #0, entitled “Synergism between natural products and antibiotics against infectious diseases”. Following, Cluster #1 labeled as “multidrug resistance efflux pump” included 48 co-cited references, and reference Poole K (2005) (Poole, 2005) in Cluster #1 had the largest co-citation counts (8 times). Moreover, Andersson DI (2010) (Andersson and Hughes, 2010) and Alekshun MN (2007) (Alekshun and Levy, 2007) in Cluster #3 had a purple outer ring with the centrality of 0.10. This indicated that these two reviews of antibiotic resistance had important impacts on the research of antibiotic adjuvants. From the average year of clusters, the earlier research on antibiotic adjuvants focused on compound synergy, which subsequently expanded toward efflux pumps and antibiotic-resistance mechanisms. More recently, the strategy of combining nanomaterials with antibiotics augmented the multifaceted approach to combat antibiotic resistance.

## 4 Discussion

Recent years have witnessed remarkable advancements in the field of antibiotic adjuvants. In this study, we identified the top 100 cited publications on antibiotic adjuvants. Bibliometric analysis of these influential publications provided focal domains and trajectories of the past research, which provided ideas and directions for further research (Arshad et al., 2020).

### 4.1 General information

The number of publications and citation counts stand as important indicators to reflect scholarly interest. Tracking the publication years of the top 100 cited papers revealed that they originated as early as 1977. During the years spanning 1977 to 1999, there was a yearly output of 0–2 original articles with innovative results. Notably, 1977 and 1978 witnessed three publications centered on clavulanic acid, which was first isolated from the culture of *Streptomyces* in 1976 (Brown et al., 1976). Clavulanic acid-amoxicillin combination as “Augmentin” was clinically approved in 1984. This catalyzed investigations into other antibiotic adjuvants. Between 1996 and 2000, studies centered on screening adjuvants from natural compounds or peptides (Vaara and Porro, 1996; Renau et al., 1999). Exponential growth in citation counts commenced in 2001, and this indicated a rapidly growing interest in antibiotic adjuvants. This time saw the identification of diverse adjuvant types. Meanwhile, several pharmaceutical companies were involved in the development of novel β-lactamase inhibitors and EPIs (Lomovskaya et al., 2001; Toney et al., 2001). 70% of the top 100 cited papers emerged after 2008, and the number of publications peaked in 2012 with 9 papers. This might be because a large amount of novel adjuvants and preclinical research are still ongoing. Given the success of β-lactam/β-lactamase inhibitor combinations and more adjuvants entering clinical trials, we believed that there was a great prospect for research on antibiotic adjuvants.

The ranking of countries and institutions in terms of publication quantity serves as important indicators to reflect the research level and impact. Analysis of the distribution in Figure 2 unveiled a concentration of productive countries in North America, European, and Asia. Notably, the United States emerged as the foremost contributor in terms of publication numbers. Concurrently, it exhibited the highest TLS within the international cooperation network. These results highlighted the great contributions and the predominant role of the United States in the development of antibiotic adjuvants. Similar results were also obtained in other bibliometric analyses of topics related to bacterial infection, such as tuberculosis, pneumonia, bacteriocins, and bacterial persisters (Chen et al., 2015; Jamali et al., 2019; Wang et al., 2019; Ju et al., 2022). As for the possible reasons, that is inseparable from significant investment by governments and pharmaceutical companies. Some works published from the United States were funded by National Institutes of Health, National Institute of Allergy and Infectious Diseases, etc. In addition, several pharmaceutical giants in the United States, like AstraZeneca and Meck & Company, also supported the research on antibiotic adjuvants (Toney et al., 2001; Hugonnet et al., 2009; Ehmann et al., 2012; Gutiérrez-Gutiérrez et al., 2016). The elevated TLS observed in North American and European countries in comparison to Asian countries underscored the former’s emphasis on international exchange and communication. This also suggested that countries from Asia need to prioritize enhanced international collaboration to bolster global academic influence.

As for institutions, one-third of the top 31 institutions with more than 2 publications were from the United States. This indicated that the United States has accounted for most of the productive institutions in the field of antibiotic adjuvants. This might partially explain why the United States consistently maintained its high quantity of publications. The degree of collaboration between institutions (DC = 0.59) surpassed that of countries (DC = 0.34). This revealed a phenomenon that cooperation and communication between institutions were predominantly confined to domestic spheres. It is imperative to transcend geographical boundaries and encourage global cooperation between academia, drug developers, and funding bodies. Further international research holds the potential to yield innovative combination strategies against antibiotic resistance.

According to the results of the author analysis, Robert E.W. Hancock was the most productive author with 5 publications as a corresponding author. These publications have been cited over 1,386 times and focused on developing AMPs or host defense peptides to enhance antibiotic action (Yeung et al., 2011; de la Fuente-Núñez et al., 2015). Laura J. V. Piddock, Xian-Zhi Li, Hiroshi Nikaido, and Olga Lomovskaya also have made great contributions, because they published more than 3 highly cited papers and they owned the most co-citations and TLS within the co-citation network. Laura J. V. Piddock has contributed 4 papers to the top 100 highly cited publications, and one of these papers has been cited over 700 times. She mainly explored the function of efflux pumps and the development of novel EPIs, especially the AcrB efflux pump (Stavri et al., 2007; Blair and Piddock, 2009; Ciusa et al., 2022). Similarly, Olga Lomovskaya focused on the activity and practical applications of EPIs (Renau et al., 1999; Lomovskaya and Bostian, 2006). Olga Lomovskaya has authored 3 publications in the list of top 100 cited publications, which have been cited over 1,145 times. She also owned co-citation counts of 66 times and great TLS of 4,073 in the author co-citation analysis. In recent years, she participated in the study of novel β-lactamase inhibitors, Vaborbactam (Sun et al., 2017). As for author co-citation analysis, Xian-Zhi Li and Hiroshi Nikaido had the largest co-citation counts (80 times and 71 times, respectively) and TLS (8,005 and 5,841, respectively). Meanwhile, they have co-authored 3 publications in the list of top 100 cited publications, and each of these publications has been cited more than 500 times. Their papers were concerned about efflux-mediated antibiotic resistance (Li et al., 2003; Li et al., 2015). Given the great contributions and high impact of these authors, we believed that these authors played pivotal roles in the field of antibiotic adjuvants. Their research accumulated important experience for the development of antibiotic adjuvants and might continue to guide cutting-edge research on antibiotic adjuvants in the future.

Journal analysis holds valuable insights for researchers to understand the authoritative journals related to antibiotic adjuvants. It also helps researchers to choose the proper journals for submitting their research. *Antimicrobial Agents and Chemotherapy* emerged as a prominent and influential journal with the largest number of publications (12) and total citation counts (4,619). This journal is also popular for researchers investigating topics related to bacterial infection, such as antibiotic-resistance of *A*. *baumannii*, *P*. *aeruginosa* biofilm, and bacterial persisters (Zhu et al., 2020; Ju et al., 2022). Despite its impact, *Antimicrobial Agents and Chemotherapy* registers a relatively low 2022 IF of 4.9 and it places in the Q2 category of the Microbiology domain. This was probably because the concentration of papers within specific topics has potentially limited its readership and citations (Bolli, 2019). This observation underlined that most researchers preferred to select reputable and authoritative journals within the microbiology field over purely focusing on IF. As a veteran journal founded in 1972, *Antimicrobial Agents and Chemotherapy* has high inclusion standards and it is still the authoritative journal in the microbiology domain. Some multi-disciplinary journals have also emerged as reputable venues for publishing high-quality studies related to antibiotic adjuvants, such as *Proceedings of the National Academy of Sciences of the United States of America*, *Plos One*, and *Nature*. Moreover, research on antibiotic adjuvants was also published in journals belonging to different disciplines, such as chemistry, pharmacology, nanotechnology, and medicine. These results revealed that multi-disciplinary researchers have increasingly concerned about the identification of novel antibiotic adjuvants or utilizing adjuvants to solve the problem of antibiotic resistance.

### 4.2 Research hotspots and frontiers

Keyword co-occurrence and references co-citation analysis provided a direct glimpse into research hotspots, the evolution of the field, and potential future trends. From the aforementioned bibliometric analysis, some of the research hotspots and frontiers in the field of antibiotic adjuvants were listed:

#### 4.2.1 Mechanism of antibiotic resistance

Resistance to antibiotics occurs through multiple molecular mechanisms, all of which can be inhibited by small molecules. Therefore, they are potential targets for antibiotic adjuvants. There are several main mechanisms of antibiotic resistance:

(1) Inactivation of antibiotics. Enzymes capable of modifying or degrading antibiotics’ key structural elements contribute to bacterial resistance. To date, tens of thousands of enzymes have been found to break down and alter various types of antibiotics, including β-lactams, aminoglycosides, chloramphenicol, and macrolides (Blair et al., 2015). A notable example is β-lactamase. It is widespread in bacterial pathogens and can hydrolyze widely used antibiotics, like penicillins, cephalosporins, clavams, carbapenems, and monobactams (Linciano et al., 2019).
(2) Increased efflux. The active transfer of antibiotics out of the cells plays an important role in the resistance of bacterial pathogens. This mode of resistance is achieved through efflux pumps and the “multidrug resistance” (MDR) efflux pumps are capable of extruding a variety of structurally diverse compounds. Since the discovery of tetracycline efflux in *E. coli* (McMurry et al., 1980), many efflux pumps have been characterized in pathogens, such as *S. aureus*, *P. aeruginosa*, *E. coli*, and mycobacteria. These efflux pumps are classified into six families based on amino acid sequence homology, and members of the resistance-nodulation-division (RND) family contribute to the most clinically relevant levels of antibiotic resistance. For instance, MexAB-OprM efflux pump of *P. aeruginosa*can export multiple different classes of antibiotics, like tetracycline, norfloxacin, and β-lactams (Li et al., 1995).
(3) Reduced permeability. Reduction of the membrane permeability can hinder the effective entrance of antibiotics, and it is the most common cause of bacterial resistance. Compared to Gram-positive bacteria, Gram-negative bacteria have a double-membrane structure and are intrinsically less permeable to several antibiotics. Many hydrophilic antibiotics cross the outer membrane by influx through outer membrane porin proteins, like the outer membrane protein F (OmpF) and outer membrane protein C (OmpC) channels of *E. coli.* Thus, alterations to the cell membrane, such as loss of porins, changes on the phospholipid and fatty acid content, as well as reduction of membrane fluidity, can prevent the entry of antibiotics into the cell and may lead to the emergence of antibiotic resistance (Mishra et al., 2012).
(4) Modification of targets. Altering the structure of targets can lead to antibiotic resistance. Modification of the molecular targets usually occurs following a site mutation of selected genes or enzymatic catalysis, like methyltransferase. Mutations of target genes reduce susceptibility to inhibition while retaining the cellular functions of the target. For example, *mecA* gene mutations in methicillin-resistant *S. aureus* encode for a β-lactam insensitive PBP2a, allowing cell wall biosynthesis to proceed (Katayama et al., 2000). In addition, modification on the ribosomal 30S or 50S subunits by methyltransferases can confer resistance to antibiotics targeting ribosomes or protein synthesis (Dzyubak and Yap, 2016). Drug resistance to macrolides, vancomycin, β-lactams, fluoroquinolones, and aminoglycosides has been achieved by target modification (Luthra et al., 2018).

#### 4.2.2 Different types of antibiotic adjuvants

There are various antibiotic adjuvants targeting the above resistance mechanisms: 1) β-lactamase inhibitors; 2) EPIs; and 3) outer membrane permeabilizers. Furthermore, several other antibiotic adjuvant strategies have also been developed, such as 4) immunomodulatory peptides and 5) nanomaterials.

(1) β-lactamase inhibitors. Currently, β-lactamase inhibitors are the only clinically approved adjuvants, and three main types of β-lactamase inhibitors have been developed. In 1976, clavulanic acid with β-lactam core was reported to inhibit serine β-lactamases (Brown et al., 1976). It became the first clinically approved and widely prescribed antibiotic adjuvant in combination with a β-lactam antibiotic. Later, the β-lactam sulfones compounds, including sulbactam and tazobactam, were discovered (Drawz and Bonomo, 2010). In 2015, avibactam with diazabicyclooctane (DBO) core in combination with ceftazidime was approved for clinical use. Afterward, another DBO compound relebactam in combination with imipenem/cilastatin was clinically approved in 2019. Moreover, vaborbactam with the scaffold of cyclic boronate esters was clinically approved when combined with meropenem in 2017. Encouraged by the success of these β-lactamase inhibitors, many other β-lactamase inhibitors are in clinical trials. In addition, novel β-lactamase inhibitors are under exploration, such as the vancomycin derivative Dipi-van and the organoselenium compound Ebselen (Chiou et al., 2015; Yarlagadda et al., 2018).
(2) EPIs. A wide variety of synthetic or natural compounds have been identified as inhibitors of bacterial efflux pumps. For example, celecoxib derivatives and natural products like reserpine and flavonoid 5′-methoxyhydnocarpin D74 inhibit the major facilitator superfamily (MFS) NorA efflux pump in *S. aureus* (Neyfakh et al., 1993; Stermitz et al., 2000; Sabatini et al., 2012). There are several peptide analogs (such as phenylalanine-arginine β-naphthylamide (PaβN)) and small molecules (such as pyranopyridines) that inhibit the AcrAB efflux pump (Lomovskaya et al., 2001; Sjuts et al., 2016). Some efflux systems are well characterized and studied with clinical relevance. However, no EPIs are clinically approved. Only MP-601,205 is currently administered as an aerosol for the treatment of patients with ventilator-associated pneumonia or cystic fibrosis (Tegos et al., 2011). This was mainly because of the diverse physiological functions that efflux pumps can perform and the unexpected toxicity due to the lack of specificity between species. Further research directions may focus on deeply characterizing the known or unknown efflux pumps in bacteria and identifying compounds that specifically inhibit efflux pumps that operate only in prokaryotic cells.
(3) Outer membrane (OM) permeabilizers. OM permeabilizers are generally cationic and amphiphilic, including charged small molecules, cationic AMPs, cationic polymers, and chelators. pentamidine is the most promising OM permeabilizers because its clinical safety and pharmacology are well known (Stokes et al., 2017). AMPs with cationic and amphiphilic properties have been extensively evaluated as antibiotic adjuvants, and colistin is the most widely studied (Lazzaro et al., 2020). Furthermore, The EPI PaβN was shown to increase membrane permeability (Lamers et al., 2013). Some other peptides, such as SLAP-S25, LABv2.1, and cationic block beta-peptide (PAS8-b-PDM12), have recently been reported to disrupt bacterial outer and cytoplasmic membranes (Clairfeuille et al., 2020; Si et al., 2020; Song et al., 2020). The development of OM permeabilizers is critically important given the difficulty of discovering new antibiotic classes for Gram-negative bacteria. Although AMPs showed promising *in vitro* and *in vivo* efficacy and safety profiles as new chemical entities, they will need to progress through clinical trials.
(4) Immunomodulatory peptides. Enhancing host defense provides alternative targets for antibiotic adjuvants. Immunomodulatory peptides are naturally occurring components of the innate immune system. they can regulate the immune response to infections. The natural cathelicidin peptide LL-37 shows weak antibacterial activity and has long been known to enhance the antimicrobial activity of the innate immune system (Hancock et al., 2012). Several synthetic immunomodulatory peptides have also been created. For example, a fragment of human lactoferricin (hLF1-11) exhibited strong infection clearance in a rabbit osteomyelitis infection model. It has entered clinical trials (Faber et al., 2005). In addition, host defense peptide IDR1018 increases wound healing and can synergize with many antibiotics (Reffuveille et al., 2014). Immunomodulatory peptides have many advantages, including a low possibility of bacterial resistance, lower toxicity, and less dosage. Extensive research into immunomodulatory peptides and their translation to clinical applications is warranted.
(5) Nanomaterials. Nanomaterials are considered as promising antibiotic adjuvants. They can enhance the activity of antibiotics through multiple mechanisms of action. Metal nanomaterials (including nano-silver and nano-copper), metal oxide nanomaterials (including copper oxide and zinc oxide nanoparticles), and non-metallic nanomaterials (including nano-selenium and nano-tellurium) have shown synergy with antibiotics in research settings (Hwang et al., 2012; Baptista et al., 2018). The application of nanomaterials as antibiotic adjuvants is promising. However, further study of their safety, efficacy, and potential integration into clinical practices is still needed.

#### 4.2.3 Future perspective

According to the results of the bibliometric analysis, antibiotic adjuvants mainly included direct resistance breaker, indirect resistance breaker, and host-modulating agents. Particularly, the direct resistance breakers, such as β-lactamase inhibitors and EPIs, are currently hotspots. This was because several β-lactamase inhibitors and EPIs have been approved for clinical application or are undergoing clinical trials. However, the emergence of extensively-drug resistant (XDR) bacteria might reduce the efficacy of these direct resistance breakers. Therefore, it is necessary to strengthen the development of indirect resistance breakers, such as OM permeabilizers. The OM permeabilizers could be used as broad-spectrum adjuvants to enhance multiple classes of antibiotics. However, the toxicity of OM permeabilizers should be taken into account, as they can also act on host cells. In addition, the low-toxicity host-modulating agents, like immunomodulatory peptides, have broad prospects for clinical application. Few studies have investigated the effect of antibiotic-adjuvant combinations on biofilm elimination. Complicated forms of infection, such as bacterial biofilms, persister bacteria, and intracellular infections, are closer to the real state of clinical infection. It is necessary to develop complicated infection models to evaluate the effects of different antibiotic adjuvants.

### 4.3 Limitations

It is essential to acknowledge the limitations in this study. First, our search strategy attempted to include as many textwords related to antibiotic adjuvants as possible. However, some relevant publications that did not use these words may have been excluded from this study. Meanwhile, we only searched the WoSCC database and this could not retrieve papers from different databases, like Scopus and Google Scholar. Second, filtering by citation counts and not excluding self-citations could inadvertently include contentious works. This might affect the objectivity of the analysis. Third, some early publications were omitted from keyword analysis due to missing author-provided keywords and keywords plus in the WoS database. Lastly, when an author was affiliated with multiple institutions/countries, this publication was counted as the work of each institution/country.

## 5 Conclusion

In conclusion, this bibliometric analysis provided a comprehensive overview of the top 100 cited publications related to antibiotic adjuvants. These highly cited papers were published between 1977 and 2020. The analysis emphasized the prominence of the USA in the field, as the USA had the most number of highly cited publications and one-third of the highly productive institutions. Notably, several pharmaceutical companies have contributed to research on antibiotic adjuvants. Distinguished researchers like Robert E.W. Hancock, Laura J. V. Piddock, Xian-Zhi Li, Hiroshi Nikaido, and Olga Lomovskaya have made remarkable contributions. Enhancing international collaboration among institutions and authors in different countries held significance. Moreover, *Antimicrobial Agents and Chemotherapy* emerged as a prominent journal for influential publications in this field. Currently, research focused on bacterial resistance mechanisms and the development of novel adjuvants. Future prospects included in-depth characterization of the known or unknown targets and exploration of more adjuvants for clinical use.

## Data Availability

The original contributions presented in the study are included in the article/Supplementary Material, further inquiries can be directed to the corresponding author.
