# Supplementary material for: Bibliometric insights into the most influential papers on antibiotic adjuvants: a comprehensive analysis
Source: Front Pharmacol. 2023 Nov 13;14:1276018. doi: 10.3389/fphar.2023.1276018 (PMC10679448; doi:10.3389/fphar.2023.1276018)
Supplement: Supplementary file 1 [file DataSheet1.PDF]

Supplementary Material

Table S1. List of the top-100 highly cited publication on antibiotic adjuvants ranked by their total citation counts in WoS database

Rank	Title	Journal	Publication Year	Publication type	2022 Impact factor*	Total citation count#
1	Mechanisms of antimicrobial peptide action and resistance	Pharmacological Reviews	2003	Review	21.1	2373
2	Three decades of beta-lactamase inhibitors	Clinical Microbiology Reviews	2010	Review	36.8	1195
3	Bacteriophage therapy	Antimicrobial Agents and Chemotherapy	2001	Review	4.9	1147
4	Attenuation of *Pseudomonas aeruginosa* virulence by quorum sensing inhibitors	Embo Journal	2003	Article	11.4	1138
5	Antimicrobial activity of the metals and metal oxide nanoparticles	Materials Science & Engineering C-Materials For Biological Applications	2014	Review	7.9	881
6	Clinically relevant chromosomally encoded multidrug	Clinical Microbiolog	2006	Review	36.8	837

	resistance efflux pumps in bacteria	y Reviews				
7	The challenge of efflux-mediated antibiotic resistance in Gram-negative bacteria	Clinical Microbiolog y Reviews	2015	Review	36.8	826
8	Clavulanic acid: a beta-lactamase-inhiting beta-lactam from *Streptomyces clavuligerus*	Antimicrobi al Agents and Chemothera py	1977	Article	4.9	715
9	Identification and characterization of inhibitors of multidrug resistance efflux pumps in *Pseudomonas aeruginosa*: novel agents for combination therapy	Antimicrobi al Agents and Chemothera py	2001	Article	4.9	696
10	Lactic acid permeabilizes gram-negative bacteria by disrupting the outer membrane	Applied and Environmen tal Microbiolog y	2000	Article	4.4	651
11	Efflux-mediated drug resistance in bacteria: an update	Drugs	2009	Review	11.5	643
12	Synergy in a medicinal plant: antimicrobial action of berberine potentiated by 5 '-methoxyhydnocarpin, a multidrug pump inhibitor	Proceedings of the National Academy of Sciences of the United States of America	2000	Article	11.1	600

13	Emerging strategies to combat ESKAPE pathogens in the era of antimicrobial resistance: a review	Frontiers In Microbiology	2019	Review	5.2	574
14	Efflux-mediated drug resistance in bacteria	Drugs	2004	Review	11.5	563
15	Synergism between natural products and antibiotics against infectious diseases	Phytomedicine	2008	Review	7.9	527
16	The antimicrobial peptides and their potential clinical applications	American Journal of Translational Research	2019	Review	2.2	506
17	Silver enhances antibiotic activity against Gram-negative bacteria	Science Translational Medicine	2013	Article	17.1	504
18	Antimicrobial host defence peptides: functions and clinical potential	Nature Reviews Drug Discovery	2020	Review	120.1	484
19	Antimicrobial peptides: key components of the innate immune system	Critical Reviews In Biotechnology	2012	Review	9	475
20	Multifunctional cationic host defence peptides and their clinical applications	Cellular and Molecular Life Sciences	2011	Review	8	463
21	Phage therapy: a renewed approach to combat antibiotic-	Cell Host & Microbe	2019	Review	30.3	406

	resistant bacteria					
22	Bacterial multidrug efflux pumps: mechanisms, physiology and pharmacological exploitations	Biochemical and Biophysical Research Communications	2014	Review	3.1	401
23	Biological activities of alpha-pinene and beta-pinene enantiomers	Molecules	2012	Article	4.6	395
24	Meropenem-clavulanate is effective against extensively drug-resistant *Mycobacterium tuberculosis*	Science	2009	Article	56.9	387
25	Engineered bacteriophage targeting gene networks as adjuvants for antibiotic therapy	Proceedings of the National Academy of Sciences of the United States of America	2009	Article	11.1	385
26	Nano-strategies to fight multidrug resistant bacteria-a battle of the Titans	Frontiers In Microbiology	2018	Review	5.2	384
27	Combination approaches to combat multidrug-resistant bacteria	Trends In Biotechnology	2013	Review	17.3	384
28	Drug tolerance in replicating mycobacteria mediated by a	Cell	2011	Article	64.5	379

	macrophage-induced efflux mechanism					
29	Bacterial efflux pump inhibitors from natural sources	Journal of Antimicrobial Chemotherapy	2007	Article	5.2	375
30	Avibactam is a covalent, reversible, non-beta-lactam beta-lactamase inhibitor	Proceedings of the National Academy of Sciences of the United States of America	2012	Article	11.1	372
31	Combinations of antibiotics and nonantibiotic drugs enhance antimicrobial efficacy	Nature Chemical Biology	2011	Article	14.8	370
32	Resistance of Gram-negative bacteria to current antibacterial agents and approaches to resolve it	Molecules	2020	Review	4.6	363
33	Modulating immunity as a therapy for bacterial infections	Nature Reviews Microbiology	2012	Review	88.1	356
34	Ceftazidime/avibactam and ceftolozane/tazobactam: second-generation beta-lactam/beta-lactamase inhibitor combinations	Clinical Infectious Diseases	2016	Review	11.8	346

35	Drug combinations: a strategy to extend the life of antibiotics in the 21 st century	Nature Reviews Microbiology	2019	Review	88.1	343
36	Bacterial multidrug efflux pumps: much more than antibiotic resistance determinants	Microorganisms	2016	Review	4.5	339
37	Phage therapy in the postantibiotic era	Clinical Microbiology Reviews	2019	Review	36.8	336
38	Synergy between essential oil components and antibiotics: a review	Critical Reviews In Microbiology	2014	Review	6.5	326
39	Bacterial resistance to antibiotics: active efflux and reduced uptake	Advanced Drug Delivery Reviews	2005	Review	16.1	321
40	Practical applications and feasibility of efflux pump inhibitors in the clinic - a vision for applied use	Biochemical Pharmacology	2006	Review	5.8	313
41	A multinational, preregistered cohort study of beta-lactam/beta-lactamase Inhibitor combinations for treatment of bloodstream infections due to extended-spectrum-beta-lactamase-producing	Antimicrobial Agents and Chemotherapy	2016	Article	4.9	307

	Enterobacteriaceae					
42	Plants as sources of new antimicrobials and resistance-modifying agents	Natural Product Reports	2012	Review	11.9	306
43	Mechanism of synergy between epigallocatechin gallate and beta-lactams against methicillin-resistant *Staphylococcus aureus*	Antimicrobial Agents and Chemotherapy	2001	Article	4.9	303
44	Enhanced antibacterial and anti-biofilm activities of silver nanoparticles against Gram-negative and Gram-positive bacteria	Nanoscale Research Letters	2014	Article	#N/A	300
45	Review on plant antimicrobials: a mechanistic viewpoint	Antimicrobial Resistance and Infection Control	2019	Review	5.5	296
46	Phytochemicals for human disease: an update on plant-derived compounds antibacterial activity	Microbiological Research	2017	Article	6.7	293
47	Antibacterial and resistance modifying activity of *Rosmarinus officinalis*	Phytochemistry	2004	Article	3.8	279
48	Alginate: current use and future	International Journal of	2016	Review	3.3	278

	perspectives in pharmaceutical and biomedical applications	Polymer Science				
49	Understanding antimicrobial activities of phytochemicals against multidrug resistant bacteria and biofilms	Natural Product Reports	2009	Review	11.9	262
50	Synergistic antibacterial effect of curcumin against methicillin-resistant *Staphylococcus aureus*	Phytomedicine	2013	Article	7.9	258
51	Natural antimicrobial peptides from bacteria: characteristics and potential applications to fight against antibiotic resistance	Journal of Applied Microbiology	2012	Review	4	253
52	Effect of farnesol on *Staphylococcus aureus* biofilm formation and antimicrobial susceptibility	Antimicrobial Agents and Chemotherapy	2006	Article	4.9	251
53	Antibiotics in the clinical pipeline at the end of 2015	Journal of Antibiotics	2017	Review	3.3	249
54	Interplay between beta-lactamases and new beta-lactamase	Nature Reviews Microbiolog	2019	Review	88.1	247

	inhibitors	y				
55	Coping with antibiotic resistance: combining nanoparticles with antibiotics and other antimicrobial agents	Expert Review of Anti-Infective Therapy	2011	Review	5.7	246
56	Ceftolozane/tazobactam: a novel cephalosporin/beta-lactamase inhibitor combination with activity against multidrug-resistant Gram-negative bacilli	Drugs	2014	Review	11.5	239
57	Clavulanic acid, a novel inhibitor of beta-lactamases	Antimicrobial Agents and Chemotherapy	1978	Article	4.9	234
58	Infections caused by resistant Gram-negative bacteria: epidemiology and management	Pharmacotherapy	2015	Review	4.1	234
59	Imipenem-relebactam and meropenem-vaborbactam: two novel carbapenem-beta-lactamase inhibitor combinations	Drugs	2018	Review	11.5	233
60	A comprehensive review on medicinal plants as antimicrobial therapeutics: potential avenues of	Metabolites	2019	Review	4.1	230

	biocompatible drug discovery					
61	Therapeutic strategies to combat antibiotic resistance	Advanced Drug Delivery Reviews	2014	Review	16.1	229
62	Insect antimicrobial peptides, a mini review	Toxins	2018	Review	4.2	229
63	Mechanisms of drug efflux and strategies to combat them: challenging the efflux pump of Gram-negative bacteria	Biochimica Et Biophysica Acta-Proteins and Proteomics	2009	Review	3.2	223
64	Use of natural antimicrobials to increase antibiotic susceptibility of drug resistant bacteria	International Journal of Food Microbiology	2010	Article	5.4	217
65	Plant antimicrobial agents and their effects onplant and human pathogens	International Journal of Molecular Sciences	2009	Review	5.6	217
66	Synthesis, optimization, and characterization of silver nanoparticles from *Acinetobacter calcoaceticus* and their enhanced antibacterial activity when combined with antibiotics	International Journal of Nanomedicine	2013	Article	8	217
67	Inhibitors of efflux pumps in *Pseudomonas*	Journal of Medicinal	1999	Article	7.3	212

	aeruginosa potentiate the activity of the fluoroquinolone antibacterial levofloxacin	Chemistry				
68	In vivo activities of amoxicillin and amoxicillin-clavulanate against *Streptococcus pneumoniae*: Application to breakpoint determinations	Antimicrobial Agents and Chemotherapy	1998	Article	4.9	210
69	An overview of antimicrobial peptides and the latest advances in their development	Expert Opinion On Biological Therapy	2017	Review	4.6	208
70	Antibiotic adjuvants: diverse strategies for controlling drug-resistant pathogens	Chemical Biology & Drug Design	2015	Article	3	207
71	Antibiotic-resistant bacteria show widespread collateral sensitivity to antimicrobial peptides	Nature Microbiology	2018	Article	28.3	206
72	Kinetic studies on the inactivation of *Escherichia coli* RTEM beta-lactamase by clavulanic acid	Biochemistry	1978	Article	2.9	205
73	Polyethyleneimine is an effective permeabilizer of Gram-negative	Microbiology-Uk	1997	Article	2.8	205

	bacteria					
74	Antibiotic efflux pumps in Gram-negative bacteria: the inhibitor response strategy	Journal of Antimicrobial Chemotherapy	2007	Article	5.2	204
75	A broad-spectrum antibiofilm peptide enhances antibiotic action against bacterial biofilms	Antimicrobial Agents and Chemotherapy	2014	Article	4.9	201
76	Multidrug efflux pumps in Gram-negative bacteria and their role in antibiotic resistance	Future Microbiology	2014	Review	3.1	201
77	Antibiotic treatment of biofilm infections	Apmis	2017	Review	2.8	200
78	D-enantiomeric peptides that eradicate wild-type and multidrug-resistant biofilms and protect against lethal *Pseudomonas aeruginosa* infections	Chemistry & Biology	2015	Article	#N/A	199
79	Bacterial efflux systems and efflux pumps inhibitors	Biochimie	2005	Review	3.9	198
80	In vitro antimicrobial activity of propolis and synergism between propolis and antimicrobial drugs	Microbiological Research	2003	Article	6.7	194

81	*Chromobacterium violaceum*: a review of pharmacological and industiral perspectives	Critical Reviews In Microbiology	2001	Review	6.5	193
82	Restoring methicillin-resistant *Staphylococcus aureus* susceptibility to beta-lactam antibiotics	Science Translational Medicine	2012	Article	17.1	193
83	Synergistic interaction between phage therapy and antibiotics clears *Pseudomonas Aeruginosa* infection in endocarditis and reduces virulence	Journal of Infectious Diseases	2017	Article	6.4	192
84	A repertoire of novel antibacterial diastereomeric peptides with selective cytolytic activity	Journal of Biological Chemistry	1997	Article	4.8	191
85	OXA-18, a class D clavulanic acid-inhibited extended-spectrum beta-lactamase from *Pseudomonas aeruginosa*	Antimicrobial Agents and Chemotherapy	1997	Article	4.9	190
86	Combined efficacy of biologically synthesized silver nanoparticles and different antibiotics against multidrug-resistant bacteria	International Journal of Nanomedicine	2013	Article	8	190

87	Pentamidine sensitizes Gram-negative pathogens to antibiotics and overcomes acquired colistin resistance	Nature Microbiology	2017	Article	28.3	189
88	Synergy and order effects of antibiotics and phages in killing *Pseudomonas aeruginosa* biofilms	Plos One	2017	Article	3.7	189
89	Antimicrobial activity of psychotropic drugs - selective serotonin reuptake inhibitors	International Journal of Antimicrobial Agents	2000	Article	10.8	188
90	In vitro activities of antibiotics and antimicrobial cationic peptides alone and in combination against methicillin-resistant *Staphylococcus aureus* biofilms	Antimicrobial Agents and Chemotherapy	2012	Article	4.9	186
91	Geraniol restores antibiotic activities against multidrug-resistant isolates from Gram-negative species	Antimicrobial Agents and Chemotherapy	2009	Article	4.9	186
92	Opportunities for natural products in 21st century antibiotic discovery	Natural Product Reports	2017	Review	11.9	186
93	Group of peptides that act synergistically with hydrophobic	Antimicrobial Agents and Chemothera	1996	Article	4.9	183

	antibiotics against gram-negative enteric bacteria	py				
94	Structure, function and inhibition of RND efflux pumps in Gram-negative bacteria: an update	Current Opinion In Microbiolog y	2009	Review	5.4	182
95	Synergistic effects between silver nanoparticles and antibiotics and the mechanisms involved	Journal of Medical Microbiolog y	2012	Article	3	179
96	Antibiotic adjuvants: rescuing antibiotics from resistance	Trends In Microbiolog y	2016	Review	15.9	179
97	Azithromycin synergizes with cationic antimicrobial peptides to exert bactericidal and therapeutic activity against highly multidrug-resistant Gram-negative bacterial pathogens	Ebiomedici ne	2015	Article	11.1	177
98	Succinic acids as potent inhibitors of plasmid-borne IMP-1 metallo-beta-lactamase	Journal of Biological Chemistry	2001	Article	4.8	176
99	The efflux inhibitor phenylalanine-arginine beta-naphthylamide (PAβN) permeabilizes the outer membrane of	Plos One	2013	Article	3.7	176

	Gram-negative bacteria					
100	Species-specific activity of antibacterial drug combinations	Nature	2018	Article	64.8	174

*The N/A represented that the journal had not been not included in the 2022 edition of JCR; #Total citation count update to April 3, 2023.
